# Synthesis of Some Novel Heterocyclic and Schiff Base Derivatives as Antimicrobial Agents

**DOI:** 10.3390/molecules201018201

**Published:** 2015-10-07

**Authors:** Mohamed E. Azab, Sameh A. Rizk, Abd El-Galil E. Amr

**Affiliations:** 1 Department of Chemistry, Faculty of Science, University of Ain Shams, Cairo 11566, Egypt; E-Mail: samehrizk2006@gmail.com; 2 Pharmaceutical Chemistry Department, Drug Exploration & Development Chair (DEDC), College of Pharmacy, King Saud University, Riyadh 11451, Saudi Arabia; E-Mail: aeamr1963@yahoo.com or aamr@ksu.edu.sa; 3 Applied Organic Chemistry Department, National Research Center, Dokki, Cairo 12622, Egypt

**Keywords:** oxirane, pyrazole, isoxazole, pyrazolopyrazine, Schiff bases, pyridones, antimicrobial activities

## Abstract

Treatment of 2,3-diaryloxirane-2,3-dicarbonitriles **1a**–**c** with different nitrogen nucleophiles, e.g., hydrazine, methyl hydrazine, phenyl hydrazine, hydroxylamine, thiosemicarbazide, and/or 2-amino-5-phenyl-1,3,4-thiadiazole, afforded pyrazole, isoxazole, pyrrolotriazine, imidazolothiadiazole derivatives **2**–**5**, respectively. Reacting pyrazoles **2a**–**c** with aromatic aldehydes and/or methyl glycinate produced Schiff’s bases **7a**–**d** and pyrazolo[3,4-b]-pyrazinone derivative **8**, respectively. Treating **7** with ammonium acetate and/or hydrazine hydrate, furnished the imidazolopyrazole and pyrazolotriazine derivatives **9** and **10**, respectively. Reaction of **8** with chloroacetic acid and/or diethyl malonate gave tricyclic compound **11** and triketone **12**, respectively. On the other hand, compound **1** was reacted with active methylene precursors, e.g., acetylacetone and/or cyclopentanone producing adducts **14a**,**b** which upon fusion with ammonium acetate furnished the 3-pyridone derivatives **15a**,**b**, respectively. Some of newly synthesized compounds were screened for activity against bacterial and fungal strains and most of the newly synthesized compounds showed high antimicrobial activities. The structures of the new compounds were elucidated using IR, ^1^H-NMR, ^13^C-NMR and mass spectroscopy.

## 1. Introduction

2,3-Diaryloxirane-2,3-dicarbonitriles are well-known as important synthetic intermediates [1,2]. The reaction of such compounds with nitrogen and carbon nucleophiles produces many biologically active heteocyclic compounds. For example, substituted pyrazoles are reported to be an important class of compounds in the agricultural and medicinal chemistry fields because of their broad spectrum biological activities [3,4,5,6] and they also have anti-cancer effects [7]. Imidazole derivatives act as potent and selective neuropeptide Y Y5 receptor antagonists, having antifungal and antibacterial activity and are used as potential tuberculostatic agents [8,9,10]. On the other hand, isooxazole derivatives have antifungal activity against *Candida albicans*, immunological and immunotropic activities [11,12,13]. Schiff bases have remarkable complex-forming properties and serve as excellent chelating ligands and have been used as analytical reagents for the spectrophotometric determination of metal ions [14,15]. For the abovementioned properties, and in continuation of our program in synthesis of biologically active heterocyclic compounds [16,17,18,19,20,21,22,23,24,25,26], we decided to use 2,3-diaryloxirane-2,3-dicarbonitrile derivatives **1a**–**c** as a key starting material for the purpose of preparing some novel heterocyclic compounds by reaction with different nitrogen and carbon nucleophiles whereby we synthesized pyrazoles, oxazoles, fused pyrazoles and pyridines, and then study their antimicrobial activity. Most of the newly synthesized compounds were screened *in vitro* for their antimicrobial activities against different strains of bacteria and fungi. Some of the compounds such as compounds **7a** and **7b** showed high antibacterial activity similar to or higher than that of the reference compounds, suggesting that they may find use as antibacterial agents. 

## 2. Results and Discussion

### 2.1. Chemistry

**Scheme 1:**
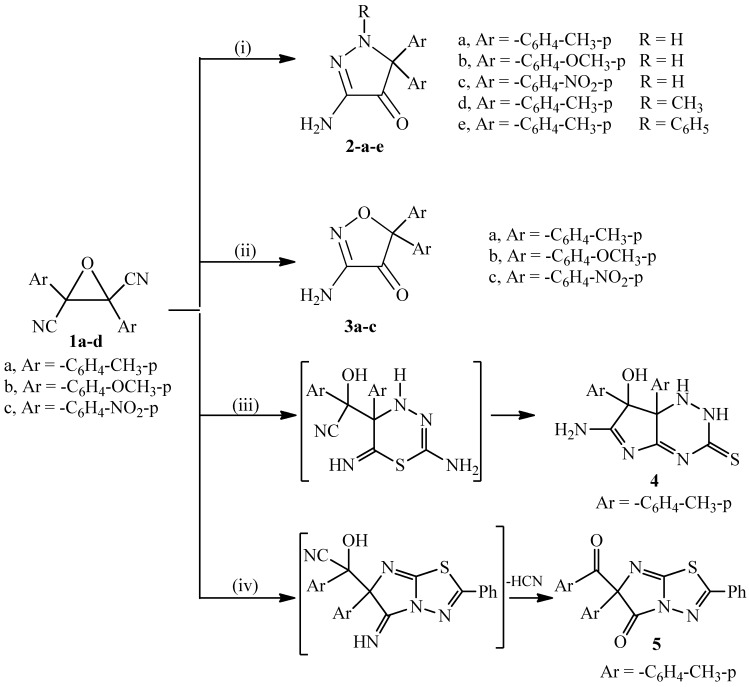
Synthetic routes for compounds **2**–**5**.

The 2,3-diaryloxirane-2,3-dicarbonitrile derivatives **1a**–**c** were allowed to react with different nitrogen binucleophiles. Thus, compounds **1a**–**c** were treated with hydrazine derivatives (hydrazine hydrate, methyl hydrazine and/or phenyl hydrazine), hydroxylamine hydrochloride, thiosemicarbazide, and/or 2-amino-5-aryl-1,3,4-thiadiazole to afford 3-amino-1-substituted-5,5-diaryl-1*H*-pyrazol-4(5*H*)-ones **2a**–**e**, 3-amino-5,5-diarylisoxazol-4(5*H*)-ones **3a**–**c**, pyrrolo[2,3-e][1,2,4]triazine-3(2*H*)-thione (**4**) and imidazo[2,1-b][1,3,4]thiadiazol-5(6H)-one (**5**), respectively (Scheme 1).

*Reagents and Conditions*: (i) N_2_H_4_/ethanol/reflux 6 h, 60%–76%; (ii) NH_2_OH/pyridine/reflux 5 h, 68%–77%; (iii) NH_2_NHCSNH_2_/ethanol/reflux, 77%; (iv) 2-amino-5-phenyl-1,3,4-thiadiazole/ethanol/ reflux 4 h, 65%.

**Scheme 2:**
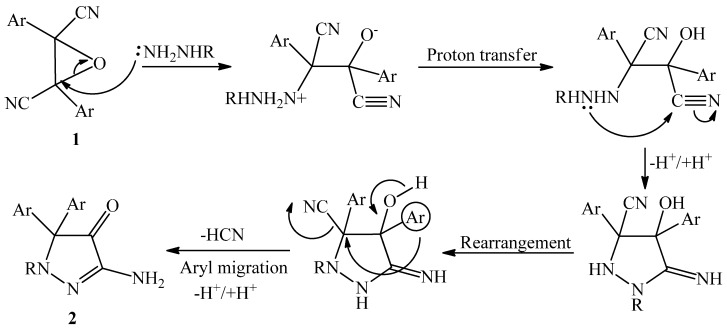
Mechanism for the formation of compound **2**.

The speculated mechanism for the formation of compound **2** is shown in Scheme 2.

**Scheme 3:**
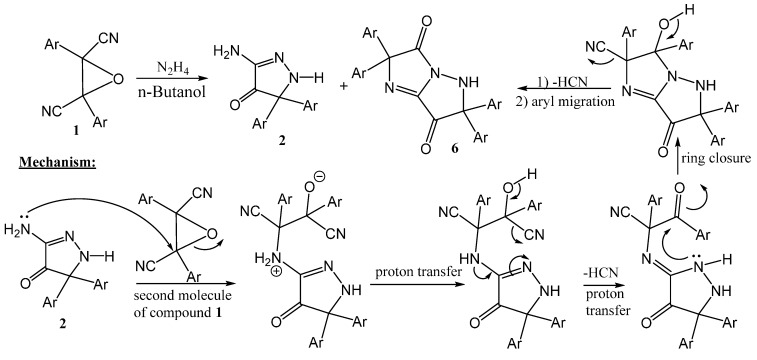
Synthetic route for compound **6**.

On the other hand, when compound **1** was reacted with hydrazine hydrate in boiling *n*-butanol, two products were isolated, one of them was the pyrazolone **2** and the other was identified to be the imidazolopyrazolone **6** (Scheme 3). The structure of **6** was elucidated from its ^1^H-NMR and ^13^C-NMR spectra, which indicate the presence of four aryl groups and two carbonyl groups. The postulated mechanism for the formation of compound **6** is shown in Scheme 3.

**Scheme 4:**
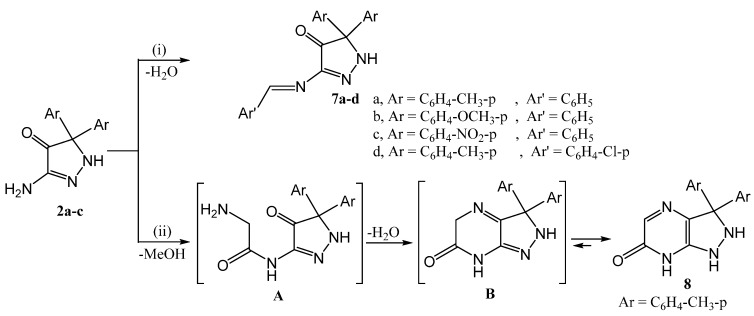
Synthetic routes for compounds **7** and **8**.

The structures of pyrazolone derivatives **2a**–**c** were supported chemically by their reaction with aromatic aldehydes (namely: benzaldehyde, and p-chlorobenzaldehyde) and/or methyl glycinate, in boiling ethanol, producing the Schiff’s bases **7a**–**d** and the pyrazolo[3,4-b]-pyrazinone derivative **8**, respectively (Scheme 4).

*Reaction Conditions*: (i) ArCHO/ethanol/reflux 4 h, 65%–75%; (ii) Methyl glycinate/ethanol/reflux 4 h, 70%.

The formation of compounds **7a**–**c** takes place through condensation reactions between the carbonyl group of the aldehyde and the amino group of compound **2** (accompanied by loss of a water molecule). The reaction of **2** with methyl glycinate takes place by the attack of the pyrazole amino group on the ester group followed by the removal of a methanol molecule forming the intermediate A, then elimination of a water molecule to form intermediate B which rearranges to the more stable form **8**. 

**Scheme 5:**
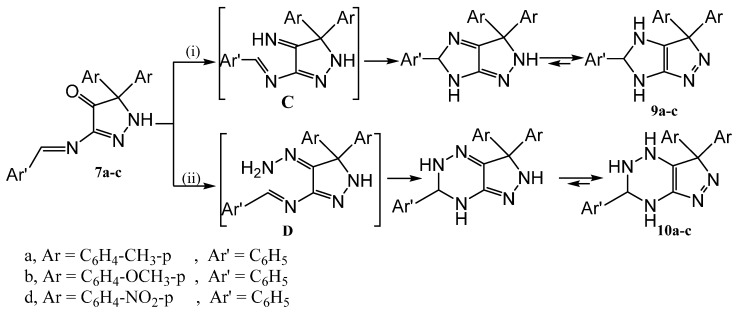
Synthetic routes for compounds **9** and **10**.

Furthermore, the Schiff's bases 7a–c were subjected to reaction with ammonium acetate (fusion at 90 °C) and/or hydrazine hydrate (in boiling ethanol), afforded the imidazolopyrazoles 9a–c and the pyrazolo[3,4-e]1,2,4-triazine derivatives 10a–c, respectively (Scheme 5).

*Reaction Conditions*: (i) Ammonium acetate/fusion at 90 °C/3 h, 66%–74%; (ii) N_2_H_4_∙H_2_O/ethanol/reflux 6 h, 68%–72%.

Compounds **9a**–**c** were formed by the formation of the imine C (by condensation of ammonia and the carbonyl group) followed by ring closure via the attack of the imino group on the C=N moiety of the Schiff's base. Similarly, formation of compounds **10a**–**c** occurs by the formation of the hydrazone D followed by ring closure via the attack of the amino group on the Schiff's base C=N.

**Scheme 6:**
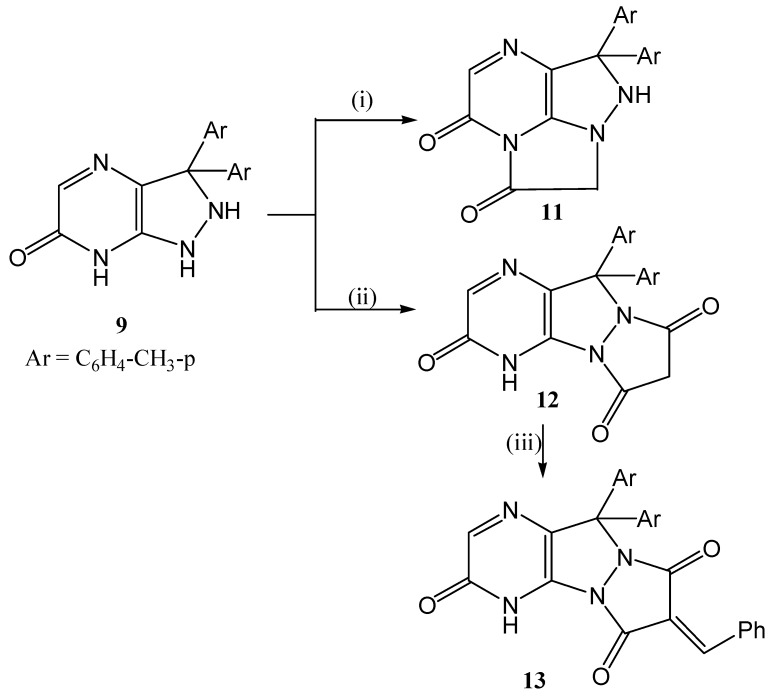
Synthetic routes for compounds **11**–**13**.

Treatment of the pyrazolopyrazinone derivative **8** with chloroacetic acid in the presence of phosphorus oxychloride furnished the tricyclic compound **11**, while reaction of diethyl malonate with **8** in boiling ethanol produced the triketone **12**, which upon refluxing with benzaldehyde in ethanol, afforded the chalcone **13** (Scheme 6).

*Reaction Conditions*: (i) ClCH_2_CO_2_H/POCl_3_/reflux 2 h, 55%; (ii) CH_2_(CO_2_C_2_H_5_)_2_/ethanol/reflux 4 h, 57%; (iii) PhCHO/ethanol/reflux 6 h, 24%.

**Scheme 7:**
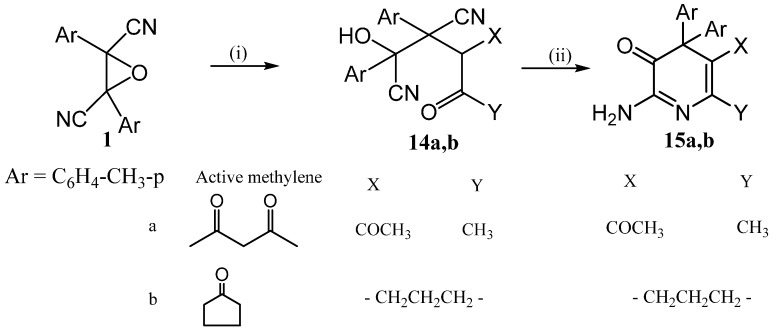
Synthetic routes for compounds **14** and **15**.

The present investigation was extended to explore the reactivity of the oxirane derivative **1a** towards some carbon nucleophiles. Thus, when compound **1a** was refluxed in ethanol with active methylene compounds (namely: acetylacetone, and/or cyclohexanone) in the presence of 50% aqueous NaOH (as a catalyst), the adducts **14a**,**b** were obtained. Cyclization of **14a**,**b**, by fusion with ammonium acetate furnished the 3-pyridone derivatives **15a**,**b** (Scheme 7).

*Reaction Conditions*: (i) Active methylene/NaOH/ethanol/reflux 3 h, 67%–70%; (ii) Ammonium acetate/fusion at 150 °C/2 h, 65%–74%.

**Scheme 8:**
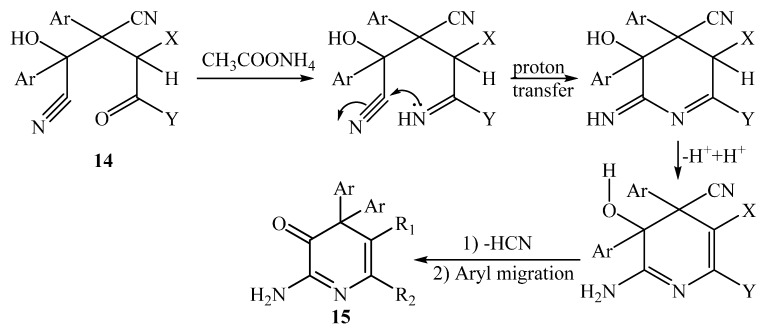
Mechanism for the formation of compounds **15a**,**b**.

A plausible mechanism for the formation of compounds **15a**,**b** is illustrated in Scheme 8.

### 2.2. Antimicrobial Activity

Most of the newly synthesized compounds were screened *in vitro* for their antimicrobial activities against different strains of bacteria and fungi. The microorganisms used were *Staphylococcus aureus* (Gram positive), *Escherichia coli*, *Pseudomonas aeruginosa* (Gram negative) and *Candida albicans* (yeast) by using the agar diffusion method [27] to select the most potent compounds.

One mg of each compound was dissolved in dimethyl sulfoxide (DMSO, 1 mL) then made up to 10 mL with sterile water to give a concentration of 100 μg/mL. The bacteria were maintained on nutrient agar media. The efficiency of the tested compounds was compared to that of ampicillin, streptomycin and nystatin. 

The agar media was incubated with the different tested microorganisms. After 24–48 h of incubation at 37 °C, DMSO showed no inhibition zones. The diameters of the inhibition zones of the tested compounds were measured. The results are presented in Table 1.

**Table 1:** Antimicrobial activity of compounds **2**–**15**.

Synthesized Compounds	StaphylococcusAureus	Escherichia Coli	Pseudomonas Aeruginosa	Candida Albicans
2a	19	20	21	15
2b	19	16	21	15
2c	17	18	16	11
4	12	12	18	12
5	12	16	18	10
6a	12	11	15	0.0
7a	18	23	22	15
7b	17	20	21	13
9a	20	17	20	21
9b	11	14	10	16
10a	13	13	0.0	15
12	14	13	0.0	11
13	16	16	19	13
14	15	17	21	12
15	13	12	0.0	11
Ampicillin	0.0	22	0.0	0.0
Streptomycin	20	21	0.0	0.0
Nystatin	0.0	0.0	0.0	22

Zone of inhibition measured in mm: no activity (0.0), very weak activity (< 7 mm), weak activity (7–10), moderate activity (11–15 mm), strong activity (> 15 mm).

The antimicrobial activity results revealed that most of the tested compounds have moderate to strong activity. The most potent compounds are **7a**, **7b** and **9a**. When these compounds were compared with the reference compounds (ampicillin, streptomycin and nystatin) we found that they have an antimicrobial activity higher or almost equal to them. On the other hand, in comparing the obtained values of antimicrobial activity for our newly synthesized Schiff bases **7a**, **7b** with similar compounds which were previously reported [28,29], we found that our compounds display higher antimicrobial activity values. 

## 3. Experimental Section 

### 3.1. General Information

All melting points are uncorrected and were determined on a Stuart electric melting point apparatus. The microanalysis were within ±0.4% of theoretical values and were carried out at the Microanalytical Centre, National Research Centre, Cairo, Egypt. IR spectra (KBr) were recorded on a FT-IR 400D infrared spectrometer (New York, USA) using the OMNIC program and are reported as frequency of absorption in cm^−1^. ^1^H-NMR spectra were recorded on a Bruker spectrophotometer (Rheinstetten, Germany) at 400 MHz using TMS as internal standard and with residual signals of the deuterated solvent δ = 7.26 ppm for CDCl_3_ and δ 2.51 ppm for DMSO-*d*_6_. ^13^C-NMR spectra were recorded on the same spectrometer at 100 MHz and referenced to solvent signals δ = 77 ppm for CDCl_3_ and δ 39.50 ppm for DMSO-*d*_6_. DEPT 135 NMR spectroscopy was used where appropriate to aid the assignment of signals in the ^1^H and ^13^C-NMR spectra. The mass spectra were recorded on a Shimadzu GCMS-QP-1000 EX mass spectrometer (Kyoto, Japan) at 70 eV using the electron ionization technique. Homogeneity of all compounds synthesized was checked by TLC which was performed on Merck 60 (Munchen, Germany) ready-to-use silica gel plates to monitor the reactions and test the purity of the new synthesized compounds. Compounds **1a**–**c** were prepared by a previously reported method [2].

### 3.2. General Procedure for the Preparation of Compounds ***2a**–**e***

An equimolar mixture of compounds **1a**–**c** (0.01 mol) and the hydrazine derivatives, namely hydrazine hydrate, methyl hydrazine and phenyl hydrazine (0.01 mol) in 50 mL ethanol was refluxed for 6 h. The solid that separated after cooling was filtered off, washed with petroleum ether (b.p 40–60 °C), dried and then crystallized from ethanol.

*3-Amino-5,5-di(4-methylphenyl)-1H-pyrazol-4(5H)-one* (**2a**). Yield 73%. m.p. 230–232 °C. IR (KBr), ν cm^−1^: 3310, 3270 (NH_2_, NH), 3056 (CH_Ar_), 1713 (CO). ^1^H-NMR (DMSO-*d*_6_): δ at 2.13 (s, 6H, 2CH_3_), 7.09–7.86 (m, 8H, ArH), 8.83 (bs, 2H, NH_2_, D_2_O exchangeable) 11.81 (s, 1H, NH D_2_O exchangeable), ^13^C-NMR (DMSO-*d*_6_) δ ppm: 23.6 (CH_3_), 75.2 (C5), 130.5, 131.7, 134.3, 135.8, 144.4 (aromatic + C=N), 191.6 (C=O). MS: *m*/*z* 279 [M^+.^] (34%). Anal. Calc. for C_17_H_17_N_3_O (279): C, 73.11; H, 6.09; N 15.05; found: C, 73.39; H, 5.87; N, 15.44.

*3-Amino-5,5-di(4-methoxyphenyl)-1H-pyrazol-4(5H)-one* (**2b**). Yield 77%. m.p. 246–248 °C. IR (KBr), ν cm^−1^: 3436, 3276 (NH_2_, NH), 3063 (CH_Ar_), 1712 (CO). ^1^H-NMR (DMSO-*d*_6_): δ 3.53 (s, 6H, 2OCH_3_), 7.43-7.82 (m, 8H, ArH), 12.12 (bs, 2H, NH_2_, D_2_O exchangeable), and 13.24 (bs, 1H, NH, D_2_O exchangeable). ^13^C-NMR (DMSO-*d*_6_) δ ppm: 59.8 (CH_3_), 77.1 (C5), 116.7, 128.4, 134.6, 143.8, 153.5 (aromatic + C=N), 191.1 (C=O). MS: *m*/*z* 311 [M^+.^] (23.7%). Anal. Calc. for C_17_H_17_N_3_O_3_ (311): C, 65.59; H, 5.46; N, 15.43; found: C 65.23, H 5.31, N 15.05.

*3-Amino-5,5-di(4-nitrophenyl)-1H-pyrazol-4(5H)-one* (**2c**). Yield 65%. m.p. 182–184 °C. IR (KBr), ν cm^−1^: 3457, 3338 (NH_2_, NH), 3065 (CH_Ar_), 1718 (CO). ^1^H-NMR(DMSO-*d*_6_): δ 7.32–7.82 (m, 8H, ArH), 11.86 (bs, 2H, NH_2_, D_2_O exchangeable) and 12.85 (bs, 1H, NH, D_2_O exchangeable). MS: *m*/*z* 341 [M^+.^] (14.8%). Anal. Calc. for C_15_H_11_N_5_O_5_ (341): C, 52.78; H, 3.22; N, 20.53; found: C, 53.02; H, 3.08, N, 20.89. 

*3-Amino-5,5-di(4-methylphenyl)-1-methyl-pyrazol-4(5H)-one* (**2d**). Yield 64%. m.p. 204–206 °C. IR (KBr), ν cm^−1^: 3324 (NH_2_), 3056 (CH_Ar_), 1722 (CO). ^1^H-NMR (DMSO-*d*_6_): δ 2.21 (s, 6H, 2CH_3_Ar), 3.36 (s, 3H, CH_3_), 7.21–7.66 (m, 8H, ArH), 11.27 (bs, 2H, NH_2_, D_2_O exchangeable). ^13^C-NMR (DMSO-*d*_6_) δ ppm: 22.7 (CH_3_), 38.5 (CH_3_), 78.6 (C5), 130.6, 132.3, 133.5, 137.9, 144.1 (aromatic + C=N), 192.0 (C=O). MS: *m*/*z* 293 [M^+.^] (12.9%). Anal. Calc. for C_18_H_19_N_3_O (293): C, 73.72; H, 6. 48; N, 14.33; found: C, 74.00; H, 6.75; N, 14.00. 

*3-Amino-5,5-di(4-methylphenyl)-1-phenyl-pyrazol-4(5H)-one* (**2e**). Yield 60%. m.p. 218–220 °C. IR (KBr), ν cm^−1^: 3321 (NH_2_), 3060 (CH_Ar_), 1720 (CO). ^1^HNMR (DMSO-*d*_6_): δ 2.26 (s, 6H, 2CH_3_), multiplet at 7.28–7.80 (m, 13H, ArH), 10.17 (bs, 2H, NH_2_, D_2_O exchangeable). ^13^C-NMR (DMSO-*d*_6_) δ ppm: 23.1 (CH_3_), 76.7 (C5), 117.8, 123.6, 126.8, 130.8, 132.1, 133.1, 135.5, 141.7, 144.6 (aromatic + C=N), 192.6(C=O). MS: *m*/*z* 355 [M^+.^] (8.9%). Anal. Calc. for C_23_H_21_N_3_O (355): C, 77.74; H, 5.91; N, 11.83; found: C, 78.05; H, 5.77; N, 11.50.

### 3.3. General Procedure for the Preparation of the Compounds ***3a**–**c***

A mixture of **1a**–**c** (0.01 mol) and hydroxylamine hydrochloride (1.03 g; 0.015 mol) in boiling pyridine (50 mL) was heated under reflux for 5h. The reaction mixture was allowed to cool, poured into ice/HCl. The product that precipitated was filtered, dried, and recrystallized from dioxane.

*3-Amino-5,5-di(4-methylphenyl)isoxazol-4(5H)-one* (**3a**). Yield 73%. m.p. 222–224 °C. IR (KBr), ν cm^−1^: 3357 (NH_2_), 3056 (CH_Ar_), 1706 (CO). ^1^H-NMR (DMSO-*d*_6_): δ 2.11 (s, 6H, 2CH_3_), 7.25–7.80 (m, 8H, Ar-H), 8.11 (bs, 2H, NH_2_, D_2_O exchangeable). ^13^C-NMR (DMSO-*d*_6_) δ ppm: 23.4 (CH_3_), 101.7 (C5), 128.3, 130.4, 134.6, 140.0, 149.2 (aromatic + C=N), 193.0(C=O). MS: *m*/*z* 280 [M^+.^] (4.6%). Anal. Calc. for C_17_H_16_N_2_O_2_ (280): C, 72.86; H, 5.71; N, 10.00; found: C, 72.57; H, 5.55; N, 10.32.

*3-Amino-5,5-di(4-methoxyphenyl)isoxazol-4(5H)-one* (**3b**). Yield 77%. m.p. 250–252 °C. IR (KBr), ν cm^−1^: 3299 (NH_2_), 3054 (CH_Ar_), 1724 (CO). ^1^H-NMR (DMSO-*d*_6_): δ 3.67 (s, 6H, 2OCH_3_), 7.23–7.89 (m, 8H, Ar-H), 9.21 (bs, 2H, NH_2_, D_2_O exchangeable). ^13^C-NMR (DMSO-*d*_6_) δ ppm: 58.2 (CH_3_), 102.2 (C5), 120.6, 129.1, 135.2, 149.5, 154.4 (aromatic + C=N), 192.1 (C=O). MS: *m*/*z* 312 [M^+.^] (15.7%). Anal. Calc. for C_17_H_16_N_2_O_4_ (312): C, 65.38; H, 5.13; N, 8.97; found: C, 65.00; H, 4.99; N 8.65. 

*3-Amino-5,5-di(4-nitrophenyl)isoxazol-4(5H)-one* (**3c**). Yield 68%. m.p. 204–206 °C. IR (KBr), ν cm^−1^: 3448 (NH_2_), 3058 (CH_Ar_), 1723 (CO). ^1^H-NMR (DMSO-*d*_6_): δ 7.16–7.74 (m, 8H, Ar-H), 8.78 (bs, 2H, NH_2_, D_2_O exchangeable). MS: *m*/*z* 342 [M^+.^] (12.8%). Anal. Calc. for C_15_H_10_N_4_O_6_ (342): C, 52.63; H, 2.92; N, 16.37; found: C, 52.99; H, 2.80; N, 16.05. 

### 3.4. 6-Amino-7-hydroxy-7,7a-di(4-methylphenyl)-7,7a-dihydro-1H-pyrrolo[2,3-e][1,2,4]-triazine-3(2H)-thione* (**4**)*

An equimolar mixture of compound **1a** (2.74 g; 0.01 mol) and thiosemicarbazide (0.91 g, 0.01 mol) in 50 mL ethanol was refluxed for 4 h. The solid that separated after cooling was filtered off, dried and then, recrystallized from ethanol. Yield 70%. m.p. 192–194 °C. IR (KBr), ν cm^−1^: 3543 (OH), 3408 (NH_2_), 3287, 3209 (NH), 3052 (CH_Ar_), 2860 (SH), 1629 (C=N). ^1^H-NMR (DMSO-*d*_6_): δ 2.19 (s, 6H, 2CH_3_), 5.63 (bs, 1H, OH, D_2_O exchangeable), 7.16–7.72 (m, 8H, ArH) 8.77 (bs, 2H, NH_2_, D_2_O exchangeable) 12.07, 12.52 (bs, 2H, 2NH, D_2_O exchangeable). ^13^C-NMR (DMSO-*d*_6_) δ ppm: 23.1 (CH_3_), 66.9, 92.4 (saturated-C), 124.7, 125.4, 126.4, 127.8, 135.3, 136.1, 140.1, 145.3, 157.2, 158.3 (Aromatic + C=N), 179.9 (C=S). MS: *m*/*z* 365 [M^+.^] (21.4%). Anal. Calc. for C_19_H_19_N_5_OS (365): C 62.47, H 5.20, N 19.18, S 8.77; found: C 62.18, H 5.40, N 18.87, S 8.45. 

### 3.5. (6-(4-Methylbenzoyl)-2-phenyl-6-(4-methyphenyl)imidazo[2,1-b][1,3,4]thiadiazol-5(6H)-one* (**5**)*

A mixture of **1a** (2.74g, 0.01 mol) and 2-amino-5-phenyl-1,3,4-thiadiazole (1.77 g; 0.01 mol) in ethanol (50 mL) was refluxed for 4h, then left to cool. The separated solid product was filtered off, dried and recrystallized from toluene-ethanol mixture. Yield 65%. m.p. 152–154 °C. IR (KBr), ν cm^−1^: 3052 (CH_Ar_), 1668, 1687 (CO), 1613 (C=N). ^1^H-NMR (DMSO-*d*_6_): δ 2.14 (s, 3H, CH_3_), 7.24–7.81 (m, 13H, ArH). ^13^C-NMR (DMSO-*d*_6_) δ ppm: 22.4 (CH_3_), 84.4 (saturated-C), 127.3, 128.1, 129.6, 130.4, 131.7, 132.6, 133.4, 134. 3,135.2, 136.3, 137.5, 138.2, 141.7, 146.9, 149.3 (aromatic + C=N), 175.8, 199.1, (C=O). MS: *m*/*z* 425 [M^+^] (5.1%). Anal. Calc. for C_25_H_19_N_3_O_2_S (425): C, 70.59; H, 4.47; N, 9.88; S, 7.53; found: C, 70.95; H, 4.28; N, 9.52; S, 7.80. 

### 3.6. General Procedure for the Preparation of Compounds ***6a**–**c***

An equimolar mixture of compounds **1a**–**c** (0.01 mol) and hydrazine hydrate (0.5 mL, 0.01 mol) in *n*-butanol (30 mL) was refluxed for 8 h. The solid that separated after cooling was filtered off, washed by petroleum ether (b.p. 40–60 °C), dried and then crystallized from *n*-butanol to afford compounds **7a**–**c**. The mother liquor (*n*-butanol) was evaporated under vacuum till dryness. The obtained solid was crystallized from ethanol producing compounds **2a**–**c**.

*2,2,6,6-Tetra(4-methylphenyl)-5,6-dihydro-2H-pyrazolo*[1,5-a]*imidazole-3,7-dione* (**6a**). Yield 30%. m.p. 296–298 °C. IR (KBr), ν cm^−1^: 3119 (NH), 3074 (CH_Ar_), 1669, 1652 (C=O). ^1^H-NMR (DMSO-*d*_6_): δ 2.14, 2.36 (s, 12H, 4CH_3_), 7.04–7.58 (m, 16H, ArH), 12.33 (bs, 1H, NH, D_2_O exchangeable). ^13^C-NMR (DMSO-*d*_6_) δ ppm: 22.4 (CH_3_), 79.9, 87.8 (saturated-C), 130.3, 131.4, 134.8, 137.1, 138.2, 140.1, 140.9, 148.8 (aromatic + C=N), 184.5, 190.8 (C=O). MS: *m*/*z* 499 [M^+.^] (7.75). Anal. Calc. for C_33_H_29_N_3_O_2_ (499): C, 79.36; H, 5.81; N, 8.42; found: C, 79.32; H, 5.83; N, 8.45. 

*2,2,6,6-Tetra(4-methoxyphenyl)-5,6-dihydro-2H-pyrazolo*[1,5-a]*imidazole-3,7-dione* (**6b**). Yield 27%. m.p. 282–284 °C. IR (KBr), ν cm^−1^: 3331 (NH), 3050 (CH_Ar_), 1680, 1666 (C=O). ^1^H-NMR (DMSO-*d*_6_): δ 3.72, 3.86 (s, 12H, 4OCH_3_), 7.54–8.20 (m, 16H, ArH), 12.51 (bs, 1H, NH, D_2_O exchangeable). ^13^C-NMR (DMSO-*d*_6_) δ ppm: 57.1 (CH_3_), 78.8, 88.1 (saturated-C), 118.7, 130.5, 133.2, 134.1, 146.3, 149.9 (aromatic + C=N), 177.8, 194.4 (C=O). MS: *m*/*z* 563 [M^+.^] (13.4%). Anal. Calc. for C_33_H_29_N_3_O_6_ (563): C, 70.34; H, 5.15; N, 7.46; found: C, 70.08; H, 4.93; N, 7.09. 

*2,2,6,6-Tetra(4-nitrophenyl)-5,6-dihydro-2H-pyrazolo*[1,5-a]*imidazole-3,7-dione* (**6c**). Yield 25%. m.p. 260–262 °C. IR (KBr), ν cm^−1^: 3276 (NH), 3073(CH_Ar_), 1685, 1669 (C=O). ^1^H-NMR (DMSO-*d*_6_): δ 7.37–7.94 (m, 16H, ArH), 12.89 (bs, 1H, NH, D_2_O exchangeable). MS: *m*/*z* 623 [M^+.^] (44.4%). Anal. Calc. for C_29_H_17_N_7_O_10_ (623): C, 55.85; H, 2.72; N, 15.73; found: C, 55.84; H, 2.70; N, 15.70.

### 3.7. General Procedure for the Preparation of the Schiff's Bases ***7a**–**d***

A mixture of compounds **2a**–**c** (0.01 mol) and aromatic aldehyde, namely benzaldehyde and/or *p*-chlorobenzaldehyde (0.01 mol) in ethanol (50 mL) was refluxed for 4 h. The solid that separated after cooling was filtered off, dried and then crystallized from ethanol.

*3-(Benzylideneamino)-5,5-di(4-methylphenyl)-1H-pyrazol-4(5H)-one* (**7a**). Yield 70%. m.p. 212–214 °C. IR (KBr), ν cm^−1^: 3221 (NH), 3056 (CH_Ar_), 1676 (CO). ^1^H-NMR (DMSO-*d*_6_): δ 2.25 (s, 6H, 2CH_3_), 6.22 (s, 1H, CH=), 7.19–7.92 (m, 13H, ArH), 11.24 (bs, 1H, NH, D_2_O exchangeable). ^13^C-NMR (DMSO-*d*_6_) δ ppm: 23.3 (CH_3_), 75.7 (saturated-C), 127.4, 128.6, 130.2, 131.4, 133.9, 134.7, 135.6, 137.9, 147.1, 152.6 (aromatic + C=N), 191.5 (C=O). MS: *m*/*z* 367 [M^+.^], 274, 194, 178, 166, 145. Anal. Calc. for C_24_H_21_N_3_O (367): C, 78.47; H, 5.72; N, 11.44; found: C, 78.81; H, 5.55; N 11.12. 

*3-(Benzylideneamino)-5,5-di(4-methoxyphenyl)-1H-pyrazol-4(5H)-one* (**7b**). Yield 75%. m.p. 226–228 °C. IR (KBr), ν cm^−1^: 3211(NH), 3058 (CH_Ar_), 1670 (CO). ^1^H-NMR (DMSO-*d*_6_): δ 3.71 (s, 6H, 2OCH_3_), 6.31 (s,1H, CH=), 7.16–7.97 (m, 13H, ArH), 11.32 (bs, 1H, NH, D_2_O exchangeable). ^13^C-NMR (DMSO-*d*_6_) δ ppm: 59.3 (CH_3_), 77.4 (saturated-C), 116.2, 128.6, 130.1, 131.5, 133.2, 134.6, 135.8, 148.5, 149.8 153.5(aromatic + C=N), 189.5(C=O). MS: *m*/*z* 399 [M^+^] (28.2%). Anal. Calc. for C_24_H_21_N_3_O_3_ (399): C, 72.18; H, 5.26; N, 10.53; found: C, 71.83; H, 5.61; N, 10.35. 

*3-(Benzylideneamino)-5,5-di(4-nitrophenyl)-1H-pyrazol-4(5H)-one* (**7c**). Yield 65%. m.p. 210–212 °C. IR (KBr), ν cm^−1^: 3278 (NH), 3080 (CH_Ar_), 1680 (CO). ^1^H-NMR (DMSO-*d*_6_): δ 6.47 (s, 1H, CH=), 7.14–8.00 (m, 13H, ArH), 10.11 (bs, 1H, NH, D_2_O exchangeable). MS: *m*/*z* 429 [M^+.^] (25.6%). Anal. Calc. for C_22_H_15_N_5_O_5_ (429): C, 67.71; H, 4.07; N, 13.16; found: C, 67.70; H, 4.02; N, 13.15. 

*3-(4-Chlorobenzylidenimino)-5,5-di(4-methylphenyl)-1H-pyrazol-4(5H)-one* (**7d**). Yield 72%. m.p. 226–228 °C. IR (KBr), ν cm^−1^: 3290 (NH), 3052 (CH_Ar_), 1672 (CO). ^1^H-NMR (DMSO-*d*_6_): δ 2.28 (s, 6H, 2CH_3_), 6.57 (s, 1H, CH=), 7.31–7.95 (m, 12H, ArH), 11.00 (bs, 1H, NH, D_2_O exchangeable). ^13^C-NMR (DMSO-*d*_6_) δ ppm: 23.0 (CH_3_), 75.1 (saturated-C), 127.7, 128.8, 130.0, 131.6, 133.5, 134.8, 135.8, 137.5, 147.4, 153.1 (aromatic + C=N), 191.8 (C=O). MS: *m*/*z* 401 [M^+.^] (33.6%), [M + 2] (11.1%). Anal. Calc. for C_24_H_20_N_3_OCl (401.5): C, 71.73; H, 4.98; N, 10.46; Cl, 8.84; found: C, 70.65; H, 4.26; N, 11.21; Cl, 8.51. 

### 3.8. 3,3-Di(4-methylphenyl)-2,3-dihydro-1H-pyrazolo[4,3-b]pyrazin-6(7H)-one *(**8**)*

A mixture of compound **2a** (2.76 g; 0.01 mol) and methyl glycinate (1 mL, 0.01 mol) in ethanol (50 mL) was refluxed for 4 h. The solid that separated out after cooling was filtered off, dried and then recrystallized from dioxane. Yield 70%. m.p. 164–166 °C. IR (KBr), ν cm^−1^: 3277–3226 (NH), 3050 (CH_Ar_), 1668 (C=O). ^1^H-NMR (DMSO-*d*_6_): δ 2.20 (s, 6H, 2CH_3_), 6.77 (s, 1H, CHpy), 7.21–7.80 (m, 8H, ArH), 6.4, 9.7 and 12.4 (3bs, 3H, 3NH, D_2_O exchangeable). ^13^C-NMR (DMSO-*d*_6_) δ ppm: 23.0 (CH_3_), 75.9 (saturated-C), 115.8, 130.1, 131.5, 134.0, 137.4, 138.7, 142.6 (aromatic + C=N), 178.4 (C=O). MS: *m*/*z* 318 [M^+.^] (23.9%). Anal. Calc. for C_19_H_18_N_4_O (318): C, 71.70; H, 5.66; N, 17.61; found: C, 71.47; H, 5.48; N, 17.28. 

### 3.9. General Procedure for the Preparation of the Compounds ***9a**–**c***

A mixture of compounds **7a**–**c** (0.01 mol) and ammonium acetate (2.31 g, 0.03 mol) was heated in an oil bath at 90 °C for 3 h. The mixture was left to cool then washed with water several times. The solid product was filtered off, dried and then, crystallized from dioxane.

*3,3-Di(4-methylphenyl)-5-phenyl-4,5-dihydroimidazo*[4,5-c]*pyrazole* (**9a**). Yield 69%. m.p. 164–166 °C. IR (KBr), ν cm^−1^: 3233, 3196 (NH), 3066 (CH_Ar_), 1604 (C=N). ^1^H-NMR (DMSO-*d*_6_): δ 2.26 (s, 6H, 2CH_3_), 5.13 (s, 1H, imidazol), 7.23–7.73 (m, 13H, ArH), 9.71 and 10.49 (bs, 2H, 2NH, D_2_O exchangeable). ^13^C-NMR (DMSO-*d*_6_) δ ppm: 22.9 (CH_3_), 67.1, 75.8 (saturated-C), 117.6, 124.5, 127.8, 128.7, 130.4, 131.9, 133.8, 137.7, 141.4, 143.3 (aromaic-C). MS: *m*/*z* 366 [M^+.^] (55.1%). Anal. Calc. for C_24_H_22_N_4_ (366): C, 78.69; H, 6.01; N, 15.30; found: C, 78.96; H, 5.75; N, 15.62. 

*3,3-Di(4-methoxyphenyl)-5-phenyl-4,5-dihydroimidazo*[4,5-c]*pyrazole* (**9b**). Yield 74%. m.p. 180–182 °C. IR (KBr), ν cm^−1^: 3280, 3208 (NH), 3050 (CH_Ar_), 1614 (C=N). ^1^H-NMR (DMSO-*d*_6_): δ 3.85 (s, 6H, 2CH_3_), 5.28 (s, 1H, imidazol), 7.12–7.92 (m, 13H, ArH), 8.88 and 9.99 (bs, 2H, 2NH, D_2_O exchangeable). ^13^C-NMR (DMSO-*d*_6_) δ ppm: 58.7 (CH_3_), 67.4, 75.2 (saturated-C), 118.5, 124.8, 127.2, 128.9, 130.7, 131.7, 133.5, 136.7, 145.8, 149.6 (aromatic-C). MS: *m*/*z* 398 [M^+^] (43.8%). Anal. Calc. for C_24_H_22_N_4_O_2_ (398): C, 72.36; H, 5.53; N, 14.07; found: C, 71.98; H, 5.75; N, 13.76.

*3,3-Di(4-nitrophenyl)-5-phenyl-4,5-dihydroimidazo*[4,5-c]*pyrazole* (**9c**). Yield 66%. m.p. 224–228 °C. IR (KBr), ν cm^−1^: 3278, 3202 (NH), 3080 (CH_Ar_), 1629 (C=N). ^1^H-NMR (DMSO-*d*_6_): δ 5.41 (s, 1H, imidazol), 7.34–8.23 (m, 13H, ArH), 10.22 and 11.09 (bs, 2H, 2NH, D_2_O exchangeable). ^13^C-NMR (DMSO-*d*_6_) δ ppm: 66.8, 75.2 (saturated-C) 120.8, 124.2, 126.1, 127.8, 129.5, 130.6, 131.7, 142.5, 146.3, 147.8 (aromatic-C). MS: *m*/*z* 428 [M^+.^] (48.2%). Anal. Calc. for C_22_H_16_N_6_O_4_ (428): C, 61.68; H, 3.74; N, 19.63; found: C, 61.89; H, 4.01; N, 19.99.

### 3.10. General Procedure for the Preparation of the Compounds ***10a**–**c***

An equimolar mixture of compounds **7a**–**c** (0.01 mol) and hydrazine hydrate (0.5 mL, 0.01 mol) in ethanol (50 mL) was refluxed for 6 h. The solid that separated out after cooling was filtered off, washed with petroleum ether (b.p. 40–60 °C), dried and then crystallized from ethanol.

*7,7-Di(4-methylphenyl)-3-phenyl-2,3,4,7-tetrahydro-1H-pyrazolo*[3,4-e][1,2,4]*triazine* (**10a**). Yield 70%. m.p. 190–192 °C. IR (KBr), ν cm^−1^: 3298, 3260, 3182 (NH), 3063 (CH_Ar_), 1630 (C=N). ^1^H-NMR (DMSO-*d*_6_): δ 2.22 (s, 6H, 2CH_3_), 5.55 (s, 1H, triazine), 7.13–7.88 (m, 13H, ArH), 9.13, 10.27 and 11.07 (bs, 3H, NH, D_2_O exchangeable). ^13^C-NMR (DMSO-*d*_6_) δ ppm: 22.5 (CH_3_), 70.2, 76.4 (saturated-C), 118.2, 124.8, 127.4, 128.9, 130.5, 131.7, 133.5, 137.2, 142.1, 145.1 (aromatic-C). MS: *m*/*z* 381 [M^+.^] (22.3%). Anal. Calc. for C_24_H_23_N_5_ (381): C, 75.59; H, 6.04; N, 18.37; found: C, 75.75; H, 5.86; N, 18.02.

*7,7-Di(4-methoxyphenyl)-3-phenyl-2,3,4,7-tetrahydro-1H-pyrazolo*[3,4-e][1,2,4]*triazine* (**10b**). Yield 72%. m.p. 181–183 °C. IR (KBr), ν cm^−1^: 3284, 3222, 3200 (NH), 3056 (CH_Ar_), 1641 (C=N). ^1^H-NMR (DMSO-*d*_6_): δ 3.79 (s, 6H, 2OCH_3_), 5.42 (s, 1H, triazine), 7.30–7.79 (m, 13H, ArH), 9.38, 10.30 and 12.00 (bs, 3H, NH, D_2_O exchangeable). ^13^C-NMR (DMSO-*d*_6_) δ ppm: 59.1 (CH_3_), 70.8, 77.3 (saturated-C), 117.8, 124.1, 126.8, 128.6, 129.9, 131.4, 133.8, 137.1, 146.2, 150.5 (aromatic-C). MS: *m*/*z* 413 [M^+^] (26.1%). Anal. Calc. for C_24_H_23_N_5_O_2_ (413): C, 69.73; H, 5.57; N, 16.95; found: C, 70.01; H, 5.71; N, 16.60; 

*7,7-Di(4-nitrophenyl)-3-phenyl-2,3,4,7-tetrahydro-1H-pyrazolo*[3,4-e][1,2,4]*triazine* (**10c**). Yield 68%. m.p. 241–243 °C. IR (KBr), ν cm^−1^: ν 3300, 3268, 3190 (NH), 3080 (CH_Ar_), 1632 (C=N). ^1^H-NMR (DMSO-*d*_6_): δ 4.8 (s, 1H, triazine), 7.31–8.28 (m, 13H, ArH), 8.49, 10.01 and 11.77 (bs, 3H, NH, D_2_O exchangeable). ^13^C-NMR (DMSO-*d*_6_) δ ppm: 69.9, 75.9 (saturated-C) 116.4, 121.4, 126.5, 127.6, 129.7, 130.7, 132.1, 138.0, 146.0, 149.9 (aromatic-C). MS: *m*/*z* 443 [M^+.^] (27.7%). Anal. Calc. for C_22_H_17_N_7_O_4_ (443): C, 59.59; H, 3.84; N, 22.12; found: C, 59.85; H, 4.07; N, 21.77. 

### 3.11. 4,4-Di(4-methylphenyl)-3,4-dihydro-7H-2a,3,5,7a-tetraazacyclopenta[c,d]indene-1,7-(2H)-dione *(**11**).*

An equimolar mixture of compound **8** (3.18 g; 0.01 mol) and chloroacetic acid (1 g; 0.01 mol) in phosphorous oxychloride (20 mL) was refluxed for 2 h. After cooling, the reaction mixture was poured onto ice/H_2_O, the solid that separated out was filtered off, washed with petroleum ether (b.p. 40–60 °C), dried and then crystallized from toluene. Yield 55%. m.p. 170–172 °C. IR (KBr), ν cm^−1^: 3222 (NH), 1677, 1655 (CO), 1613 (C=N). ^1^H-NMR (DMSO-*d*_6_): δ 2.16 (s, 6H, 2CH_3_), 4.17 (s, 2H, CH_2_), 6.72 (s, 1H, pyrazine), 7.44–7.76 (m, 8H, ArH), 8.72 (bs, 1H, NH, D_2_O exchangeable). ^13^C-NMR (DMSO-*d*_6_) δ ppm: 23.2 (CH_3_), 57.1, 76.8 (sp3-C), 117.4, 127.8, 130.7, 131.9, 134.1, 137.6, 145.1 (aromatic + sp2-C), 160.2, 170.1 (C=O). MS: *m*/*z* 358 [M^+^] (43.7%). Anal. Calc. for C_21_H_18_N_4_O_2_ (358): C, 70.39; H, 5.03; N, 15.64; found: C, 70.08; H, 4.81; N, 16.01.

### 3.12. 10,10-Di(4-methylphenyl)-4,10-dihydro-3H,6H-pyrazolo[1′,2′:1,2]pyrazolo[3,4-b]-pyrazine-3,6,8-(7H)-trione *(**12**)*

A mixture of compound **8** (3.18 g; 0.01 mol) and diethyl malonate (2.4 mL; 0.015 mol) in ethanol (50 mL) was refluxed for 4 h. The solid that separated out after cooling was filtered off, washed with petroleum ether (b.p. 40–60 °C), dried and then recrystallized from ethanol. Yield 57%. m.p. 224–226 °C. IR (KBr), ν cm^−1^: 3245 (NH), 1691, 1672, 1659 (CO), 1620 (C=N), ^1^H-NMR (DMSO-*d*_6_): δ 2.15 (s, 6H, 2CH_3_), 4.51(s, 2H, COCH_2_CO), 6.81 (s, 1H, pyrazine), 7.32–7.83 (s,8H, ArH), 10.14 (bs, 1H, NH, D_2_O exchangeable). ^13^C-NMR (DMSO-*d*_6_) δ ppm: 23.0 (CH_3_), 49.4, 76.0 (saturated-C), 117.0, 127.4, 130.0, 131.2, 134.7, 138.0, 145.9 (aromatic + C=N), 161.3, 170.9, 175.3 (C=O). MS: *m*/*z* 386 [M^+^] (44.4%). Anal. Calc. for C_22_H_18_N_4_O_3_ (386): C, 68.39; H, 4.66; N, 14.51; found: C, 68.07; H, 4.88; N, 14.22. 

### 3.13. 7-Benzylidene-10,10-di(4-methylphenyl)-4,10-dihydro-3H,6H-pyrazolo[1',2':1,2]-pyrazolo[3,4-b]pyrazine-3,6,8(7H)-trione *(**13**)*

An equimolar mixture of compound **12** (3.86 g; 0.01 mol) and benzaldehyde (1.06 mL; 0.01 mol) in ethanol (50 mL) was refluxed for 6 h. The solid that separated after cooling was filtered off, dried and then crystallized from *n*-butanol. Yield 24%. m.p. 266–268 °C. IR (KBr), ν cm^−1^: 3290 (NH), 1687, 1677, 1666, (CO), 1626 (C=N). ^1^H-NMR (DMSO-*d*_6_): δ 2.26 (s, 6H, 2CH_3_), 6.44, 6.87 (bs, 2H, benzylic and pyrazine), 7.27–7.87 (m, 13H, ArH), 9.82 (bs,1 H, NH, D_2_O exchangeable). ^13^C-NMR (DMSO-*d*_6_) δ ppm: 23.4 (CH_3_), 76.5 (saturated-C), 117.8, 125.1, 126. 4, 127.7, 128.9, 130.0, 131.2, 133.0, 134.7, 136.3, 138.0, 145.9, 150.7 (aromatic + C=N), 160.5, 171.2, 173.7 (C=O). MS: *m*/*z* 474 [M^+^] (77.6%). Anal. Calc. for C_29_H_22_N_4_O_3_ (474): C, 73.42; H, 4.64; N, 11.81; found: C 73.05; H, 4.35; N, 12.13. 

### 3.14. General Procedure for the Preparation of Compounds **14a**,**b**

An equimolar mixture of compound **1a** (2.74 g; 0.01 mol) and an active methylene precursor, e.g., acetylacetone and/or cyclopentanone (0.01 mol) and aqueous NaOH (50%, 8 mL) in ethanol (50 mL) was refluxed for 3 h and left overnight. The reaction mixture was poured into ice/HCl. The solid so formed filtered off, washed with water, dried and then crystallized from ethanol.

*3,3-Diacetyl-1,2-di(4-methylphenyl)-1,2-dicyanopropanol* (**14a**). Yield 70%. m.p. 220–222 °C. IR (KBr), ν cm^−1^: 3380 (OH), 3062 (CH_Ar_), 2240, 2220 (CN), 1688 (CO). ^1^H-NMR (DMSO-*d*_6_): δ 2.19 (s, 6H, 2CH_3_), 2.43 (s, 6H, 2CH_3_), 4.29 (s, 1H, COCHCO), 5.55 (bs, 1H, OH, D_2_O exchangeable), 7.25–7.84 (m, 8H, ArH). ^13^C-NMR (DMSO-*d*_6_) δ ppm: 22.5, 30.2, (CH_3_), 36.0, 63.1, 78.2 (saturated-C), 116.8, 120.0 (CN), 125.3, 127.1, 129.6, 131.2, 134.6, 136.8, 141.1, 146.9 (aromatic-C), 200.2 (C=O). MS: *m*/*z* 374 [M^+.^] (87.4%). Anal. Calc. for C_23_H_22_N_2_O_3_ (374): C, 73.80; H, 5.88; N, 7.48; found: C, 74.15; H, 5.67; N, 7.16. 

*1,2-Dicyano-1,2-di(4-methylphenyl)-2-(2-oxocyclopentyl)ethanol* (**14b**). Yield 67%. m.p. 192–194 °C. IR (KBr), ν cm^−1^: 3367 (OH), 3051 (CH_Ar_), 2950 (CH_Ali_), 2243, 2221 (CN), 1677 (CO), ^1^H-NMR (DMSO-*d*_6_): δ 1.79–2.01 (m, 6H, CHcyclopent.), 2.13 (s, 6H, 2CH_3_), 2.31 (s, 1H, CHcyclopent), 5.88 (bs, 1H, OH, D_2_O exchangeable), 7.15–7.78 (m, 8H, ArH). ^13^C-NMR (DMSO-*d*_6_) δ ppm: 15.4, 21.5, 22.7, 36.9, 42.3, 52.0, 77.2 (saturated-C), 116.1, 119.2 (CN), 125.5, 127.4, 129.9, 130.9, 133.8, 136.3, 141.1, 145.2 (aromatic-C), 209.0 (C=O). MS: *m*/*z* 358 [M^+.^] (77.7%). Anal. Calc. for C_23_H_22_N_2_O_2_ (358): C, 76.09; H, 6.15; N 7.82; found: C, 75.73; H, 5.89; N, 8.14.

### 3.15. General Procedure for the Preparation of the Compounds ***15a**,**b***

Compounds **14a**,**b** (0.01 mol) and ammonium acetate (2.31 g, 0.03 mol) were mixed thoroughly and fused in an oil bath at 150 °C for 2 h. Left to cool, then washed with water several times. The solid product was dried and recrystallized from benzene.

*5-Acetyl-2-amino-6-methyl-4,4-di(4-methylpheny)pyridin-3(4H)-one* (**15a**). Yield 74%. m.p. 210–212 °C. IR (KBr), ν cm^−1^: 3285 (NH_2_). 1691, 1665 (CO), 1630 (C=N), ^1^H-NMR (DMSO-*d*_6_): δ 2.14 (s, 6H, 2CH_3_), 2.41 (s, 3H, CH_3_), 2.77(s, 3H, CH_3_), 7.44–7.83 (m, 8H, ArH), 9.42 (bs, 2H, NH_2_, D_2_O exchangeable). ^13^C-NMR (DMSO-*d*_6_) δ ppm: 22.5, 23.6, 28.5 (CH_3_), 52.0 (saturated-C), 128.7, 130.0, 131.9, 133.2, 137.0, 140.9, 145.6 (aromatic + C=N), 192.7, 198.00 (C=O). MS: *m*/*z* 346 [M^+^] (41.8%). Anal. Calc. for C_22_H_22_N_2_O_2_ (346): C, 76.30; H, 6.36; N, 8.09; found: C, 76.60; H, 6.00; N, 8.41. 

*2-Amino-4,4-(4-methylpheny)-4,5,6,7-tetrahydro-3H-cyclopenta[b]pyridin-3-one* (**15b**). Yield 65%. m.p. 176–178 °C. IR (KBr) ν cm^−1^: 1613 (C=N), 1685 (CO), 3272 (NH). ^1^H-NMR (DMSO-*d*_6_): δ 1.79 (q, 2H, CH_2_-cyclopentane), 2.17–2.41 (m, 10H, 2CH_3_, 2CH_2_-cyclopent.), 7.32–7.73 (m, 8H, ArH), 9.66 (bs, 2H, NH_2_, D_2_O exchangeable). ^13^C-NMR (DMSO-*d*_6_) δ ppm: 20.8, 22.4, 26.9, 47.1, 54.0 (saturated-C), 129.2, 130.4, 131.6, 133.8, 137.4, 140.9, 141.8 (aromatic + C=N), 192.0 (C=O). MS: *m*/*z* 330 [M^+^] (37.6%). Anal. Calc. for C_22_H_22_N_2_O (330): C, 80.00; H, 6.67; N, 8.48; found: C, 79.97; H, 6.63; N, 8.48.

## 4. Conclusions 

In the present work, a series of some Schiff bases and novel fused heterocyclic derivatives **2**–**15** were synthesized using 2,3-diaryloxirane-2,3-dicarbonitriles **1a**–**c** as starting materials. Some of newly synthesized compounds were screened against bacterial and fungal strains and most of the newly synthesized compounds showed high antimicrobial activities. The structures of the new compounds were elucidated using IR, ^1^H-NMR, ^13^C-NMR and mass spectroscopy.

## References

[1] Muller A.J., Nishiyama K., Griffin G.W., Ishikawa K., Gibson D.M. (1982). Reductive condensation of methyl arylglyoxylates. Direct synthesis of 2,3-bis(carbomethoxy)stilbene oxides and related systems. J. Org. Chem..

[2] Li Z., Xu J., Niu P., Liu C., Yang J. (2012). Direct synthesis of 2,3-diaryloxirane-2,3-dicarbonitriles from aroyl chlorides using potassium hexacyanoferrate(II) as an eco-friendly cyanide source. Tetrahedron.

[3] Daidone G., Maggio B., Plescia S., Raffa D., Musiu C., Milia C., Perra G., Marongiu M.E. (1998). Antimicrobial and antineoplastic activities of new 4-diazopyrazole derivatives. Eur. J. Med. Chem..

[4] Singh N., Sangwan N.K., Dhindsa K.S. (2000). Synthesis and fungitoxic activity of 5-aryl-1-formyl-4,5-dihydro-3-(2-hydroxyphenyl)-1H-pyrazoles and their complexes. Pest Manag. Sci..

[5] Daidone G., Raffa D., Plescia F., Maggio B., Roccaro A. (2002). Synthesis of pyrazole-4-carbohydrazide derivatives of pharmaceutical interest. Arkivoc.

[6] Migliara O., Plescia S., Diana P., di Stefano V., Camarda L., Dall’Olio R. (2004). Synthesis and pharmacological evaluation of 7-substituted 1-ethyl-3,4,10-trimethyl-1,10-dihydro-11*H*-pyrazolo[3,4-*c*][1,6]benzodiazocin-11-one. A new ring system. Arkivoc.

[7] Bouabdallah I., M’Barek L.A., Zyad A., Ramdani A., Zidane I., Melhaoui A. (2006). Anticancer effect of three pyrazole derivatives. Nat. Prod. Res..

[8] Sato N., Jitsuoka M., Ishikawa S., Nagai K., Tsuge H., Ando M., Okamoto O., Iwaasa H., Gomori A., Ishihara A. (2009). Discovery of substituted 2,4,4-triarylimidazoline derivatives as potent and selective neuropeptide Y Y5 receptor antagonists. Bioorg. Med. Chem. Lett..

[9] Plachta D.A., Baranowski A.M., Laudy A.E., Starosciak B.J., Kleps J. (2007). Synthesis of 1-{4-[4-(adamant-1-yl)phenoxymethyl]-2-(4-bromophenyl)-1,3-dioxolan-2-ylmethyl}imidazole with expected antifungal and antibacterial activity. Acta Pol. Pharm. Drug Res..

[10] Owawiak J., Olender D., Zwolska Z., Augustynowicz-Kopec E., Zaprutko L. (2008). Synthesis of 2,3-dihydro-7-nitroimidazo[5,1-b]oxazoles as potential tuberculostatic agents. Acta Pol. Pharm. Drug Res..

[11] Sharma P.C., Sharma S.V., Jain S., Singhd D., Suresh B. (2009). Synthesis of some new isoxazoline derivatives as possible anti-*candida* agents. Acta Pol. Pharm. Drug Res..

[12] Maczynski M., Zimecki M., Taraszkiewicz M., Ryng S. (2008). Synthesis, immunological activity and computational study of 5-amino-3-methyl-4-isoxazolecarboxylic acid semicarbazides and thiosemicarbazides. Acta Pol. Pharm. Drug Res..

[13] Maczynski M., Zimecki M., Ryng S. (2008). A new class of isoxazole derivatives: The m 1–9 series of compounds with immunotropic activity. Acta Pol. Pharm. Drug Res..

[14] Jain M., Nehra S., Trivedi P.C., Singh R.V. (2002). Nematicidal, fungicidal and bactericidal activities of manganese(II) complexes with heterocyclic sulphonamide imines. Metal Based Drugs.

[15] Patil R.M. (2007). Synthetic, structural and biological properties of binuclear complexes with some schiff bases. Acta Pol. Pharm. Drug Res..

[16] Rizk S.A., El-Hashash M.A., Mostafa K.K. (2008). Utility of β-aroyl acrylic acids in heterocyclic synthesis. Egypt. J. Chem..

[17] Rizk S.A., EL-Hashash M.A., Aburzeza M.M. (2011). Utility of *p*-acetamidobenzoyl prop-2-enoic acid in the synthesis of new α-amino acids and using them as building blocks in heterocyclic synthesis. Egypt. J. Chem..

[18] Rizk S.A. (2011). Utility of *E*-1-(4-acetamidobenzoyl)-2-oxirane carboxylic acid in synthesis some fused heterocycles and spiro compounds. Amer. J. Chem..

[19] Azab M.E., Kassab E.A., El-Hashash M.A., Ali R.S. (2009). Synthesis and antibacterial activity of some new 4(3H)quinazolin-4-one derivatives. Phos. Sulf. Silicon.

[20] Azab M.E., Youssef M.M., El-Bordany E.A. (2013). Synthesis and antibacterial evaluation of novel heterocyclic compounds containing a sulfonamido moiety. Molecules.

[21] Khamees H., Jaleel G.A.A., Azab M.E., Mohamed G.A.M., Abdel-Aziz T.A., Eyada H.A. (2013). Synthesis, characterization, anticancer, analgesic, and antiinflammatory activities of hitherto unknown thiazolo[3,2-a]pyridine and thiazolo[3,2-a]-1,8-naphthyridine derivatives. J. Atoms Mol..

[22] Azab M.E., Amr A.E. (2015). Synthesis of chiral linear and macrocyclic candidates: III. Synthesis and antimicrobial activity of linear tetrapeptide and macrocyclic pentapeptide Schiff bases. Russ. J. Gen. Chem..

[23] Said A.S., Amr A.E., El-Sayed H.A., Al-Omar M.A., Abdalla M.M. (2015). Synthesized of some heterocyclic systems and their nucleoside of potent anti-inflammatory activities. Int. J. Pharm..

[24] Fayed A.A., Al-Harb N., Amr A.E., Kalmoush A.A., Shadid K.H., Flefel E.M. (2014). Synthesis, reactions, and pharmacological evaluations of some novel pyridazolopyridiazine candidates. J. Het. Chem..

[25] Khalifa N.M., Al-Omar M.A., Amr A.E., Baiuomy A.R., Abdel Rahman R.F. (2015). Synthesis and biological evaluation of some novel fused thiazolo[3,2-a]pyrimidines as potential analgesic and antiinflammatory agents. Russ. J. Bioorg. Chem..

[26] Ouf N.H., Amr A.E., Sakran M.I. (2015). Anticancer activity of some newly synthesized pyrano[2,3-d][1,2,3]triazine derivatives using 1-(7-hydroxy-2,2-dimethyl-chroman-6-yl)ethanone as synthon. Med. Chem. Res..

[27] Carson C.F., Riley T.V. (1995). Antimicrobial activity of the major components of the essential oil of *Melaleuca alternifolia*. J. Appl. Bact..

[28] Alam M.S., Lee D.U., Bari L. (2014). Antibacterial and cytotoxic activities of Schiff base analogues of 4-aminoantipyrine. J. Korean Soc. Appl. Biol. Chem..

[29] Cheng Q., Xu X., Wang Q., Zhang L., Lin Q., Zhang J., Yang X. (2009). Synthesis and antibacterial activities of novel pyrazole Schiff bases and metal complexes. Chinese J. Org. Chem..

